# NKILA, a prognostic indicator, inhibits tumor metastasis by suppressing NF-κB/Slug mediated epithelial-mesenchymal transition in hepatocellular carcinoma

**DOI:** 10.7150/ijbs.39582

**Published:** 2020-01-01

**Authors:** Ronggao Chen, Qiyang Cheng, Kwabena Gyabaah Owusu-Ansah, Guangyuan Song, Donghai Jiang, Lin Zhou, Xiao Xu, Jian Wu, Shusen Zheng

**Affiliations:** 1 Department of Surgery, Division of Hepatobiliary and Pancreatic Surgery, First Affiliated Hospital, School of Medicine, Zhejiang University, Hangzhou 310000, China.; 2 NHFPC Key Laboratory of Combined Multi-organ Transplantation, Hangzhou 310000, China.; 3 Key Laboratory of the diagnosis and treatment of organ Transplantation, CAMS.; 4 Key Laboratory of Organ Transplantation, Zhejiang Province, Hangzhou 310003, China.; 5 Collaborative innovation center for Diagnosis treatment of infectious diseases, Hangzhou 310000, China.

**Keywords:** LncRNA-NKILA, NF-κB, Hepatocellular carcinoma, Metastasis, Epithelial to mesenchymal transition

## Abstract

The metastasis of hepatocellular carcinoma (HCC) is one of the major obstacles hindering its therapeutic efficacy, leading to low surgical resection rate, high mortality and poor prognosis. Accumulating evidence has shown that both long noncoding RNA (lncRNA) and NF-κB play vital roles in the regulation of cancer metastasis. However, the clinical significance and biological function of NKILA (NF-κB interacting lncRNA) and its interaction with NF-κB in HCC remain unknown. In this study, we demonstrated that NKILA was down-regulated in HCC tissues and cell lines, and decreased NKILA expression was significantly associated with larger tumor size and positive vascular invasion in HCC patients. NKILA reduction was an independent risk factor of HCC patients' poor prognosis, and the 5-year overall survival (OS) rates of patients with low and high NKILA expression were 15.6% and 60.0%, respectively. Moreover, NKILA inhibits migration and invasion of HCC cells both *in vitro* and *in vivo*. Mechanistically, NKILA prevents Slug/epithelial to mesenchymal transition (EMT) pathway via suppressing phosphorylation of IκBα, p65 nuclear translocation and NF-κB activation. In conclusion, these results indicate that NKILA might serve as an effective prognostic biomarker and a promising therapeutic target against HCC metastasis.

## Introduction

Hepatocellular carcinoma (HCC) is an urgent public health problem. Worldwide, HCC is the third leading cause of cancer-related mortality 1. In China, HCC is one of the most common malignancies and the top cause of cancer-related death in men under 60 2. The metastasis of HCC is one of the major obstacles hindering its therapeutic efficacy, leading to low surgical resection rate, high mortality and poor prognosis. The median survival and one-year survival rate of patients with extra-hepatic metastasis from HCC are only 4.9-7 months and 21.7%-24.9% 3, 4. Therefore, exploring the molecular mechanism of HCC metastasis is urgently needed to develop new diagnostic and therapeutic strategies to improve the situation.

Long noncoding RNAs (lncRNAs) refer to a class of transcripts longer than 200 nucleotides without protein-coding ability 5. Currently, many studies have revealed that lncRNAs are frequently dysregulated in multiple cancers and involved in various biological functions, such as proliferation, metastasis, apoptosis and drug resistance 6-8. Particularly, extensive evidence has shown that lncRNAs have an important part in regulating HCC metastasis. For instance, lncRNA miR503HG inhibits HCC metastasis by regulating the HNRNPA2B1/NF-κB pathway 9. lncRNA ZFAS1 promotes metastasis via sponging miR-150 and up-regulating ZEB1 in hepatocellular carcinoma 10. Researches focusing on lncRNA provide a new perspective for further elucidation of the molecular mechanism underlying HCC metastasis.

Nuclear factor-κB (NF-κB) is a family of transcription factors critical in regulating immune and inflammatory responses, cancer initiation and progression 11, 12. Normally, NF-κB p65/p50 components associated with IκB are retained in the cytoplasm as an inactive state. Upon stimulation, IκB kinase complex (IKK) is activated and phosphorylates IκBα, leading to its ubiquitination and subsequent degradation. Consequently, p65/p50 heterodimer is released and translocated to the nucleus, regulating the expression of genes it binds to 13, 14. To date, accumulating studies have shown that NF-κB plays vital roles in the regulation of epithelial-mesenchymal transition (EMT) and cancer metastasis 15, 16. A better understanding of the specific function of NF-κB pathway on HCC metastasis is essential to accelerate the development of novel anti-metastasis therapies. NF-κB interacting lncRNA (NKILA) has been reported to inhibit NF-κB pathway and suppress tumor metastasis in breast cancer 17 and non-small cell lung cancer 18. However, the precise role of NKILA and its interaction with NF-κB in HCC remain unclear.

Therefore, we detected the expression pattern of NKILA in HCC tissues and investigated the function and mechanism of NKILA on HCC cell biological behavior. Herein, we demonstrate that NKILA is down-regulated in HCC tissues and cell lines, and positively correlates with HCC patients' overall survival. Moreover, NKILA inhibits metastasis of HCC cells both *in vitro* and *in vivo*. Mechanistically, NKILA prevents EMT via the inhibition of NF-κB/Slug pathway. These results indicate that NKILA might serve as an effective prognostic biomarker and a promising therapeutic target against HCC metastasis.

## Methods

### Patient specimens

139 pairs of tumor and corresponding adjacent normal tissues were collected from HCC patients who underwent curative hepatectomy at the First Affiliated Hospital, Zhejiang University School of Medicine from 2011 to 2013. All included individuals received no chemotherapy or radiation therapy before surgery. The end point of follow up was Dec. 2018. Written informed consent was obtained from all subjects, and this study was approved by the ethics committee of the First Affiliated Hospital, Zhejiang University School of Medicine.

### Cell culture

All the cells were purchased from China Center for Type Culture Collection (CCTCC). Human hepatocellular carcinoma cells (SMMC-7721, SK-hep-1, HCC-LM3, Huh-7) and human immortalized normal hepatocytes (L-02) 19 were maintained in DMEM (Sigma, USA), MEM (Gibco, USA), or RPMI 1640 (Sigma, USA) containing 10% fetal calf serum (Gibco, USA) at 37 °C in a humidified incubator (Thermo Scientific, USA) with 5% CO2.

### RNA isolation and qRT-PCR

Total RNA was extracted from tissue samples or cell lines using TRIzol reagent (Invitrogen, USA) following the manufacturer's protocol. Then the reverse transcription was performed using the iScript™ cDNA Synthesis Kit (Bio-Rad, China) according to the manufacturer's instruction. The expression level of NKILA was measured by SYBR green qRT-PCR assay (Takara, China). The primers (Tsingke, China) were as follows: NKILA forward 5'-AACCAAACCTACCACAACG-3' and reverse 5'-ACCACTAAGTCAATCCCAGGTG-3'; GAPDH forward 5'- CCTGGTATGACAACGAATTTG-3' and reverse 5'-CAGTGAGGGTCTCTCTCTTCC-3'.

### Lentivirus transfection

SMMC-7721 and HCC-LM3 were transfected with lentiviral vector expressing NKILA or empty vector (Genechem, China) following standard procedure. 48 h after transfection, the cells were treated with puromycin (Sigma, USA) at a final concentration of 4 μg/ml for 2 weeks to establish stable expression cell lines.

### Cell proliferation assays

Cell Counting Kit-8 (Dojindo Laboratories, Japan) was used to detect cell proliferation. Cells (1×10^4^) were seeded into 96-well plates and after one night of incubation, determined at 450 nm using a microplate reader (Bio-Rad, CA) for four consecutive days.

### Transwell migration and invasion assays

Migration assays were performed using 24-well Transwell chambers (Corning, USA). 5×10^4^ cells resuspended in 200 μL serum-free medium were seeded to the upper chamber. The lower chamber was filled with 500 μL medium containing 10% FBS. After incubation for 24 h, cells on the membrane were stained with crystal violet (Thermo Scientific, USA) and then were examined and photographed using an inverted microscope (Leica, USA). Invasion assays were performed according to the same procedures except that transwell chamber was coated with 50 μL matrigel and incubation for 48 h.

### *In vivo* metastasis assay

A total of 10^6^ cells in 100 μL PBS were injected into each athymic nude mice through tail veins to establish *in vivo* metastasis models. After 6 weeks, the animals were sacrificed and the lungs were harvested and fixed in formalin. After embedded with paraffin, slides were prepared and underwent hematoxylin and eosin (H&E) staining. Afterwards, the stained slides were examined and photographed under microscopy. The animal experiments were approved by the Ethics Committee for Laboratory Animals of the First Affiliated Hospital, Zhejiang University School of Medicine.

### Western blot analysis and antibodies and subcellular extraction

The detailed procedure has been described in our previous study 20. Briefly, proteins were isolated with RIPA lysis buffer (Servicebio, China) and quantified with BCA Protein assay kit (Thermo Scientific, USA). Then equal amounts of proteins were fractionated on 10% SDS-PAGE gels (Invitrogen, USA) and transferred to PVDF membranes (Millipore, USA). After blocked with skim milk, the membranes were incubated with various primary antibodies at 4 °C overnight, and then incubated with corresponding secondary antibodies for 1h. Subsequently, the bands were visualized using ECL kits (Abcam, USA). The primary antibodies (Cell Signaling Technology, USA) were as follows: E-Cadherin (#3195), N-Cadherin (#13116), Vimentin (#5741), Slug (#9585), β-actin (#4970), p-IKKα/β (#2697), p-IκBα (#2859), IκBα (#4814), p65 (#8242), p-p65 (#3033), Lamin-A (#86846).

Subcellular fractions were performed using the Nuclear and Cytoplasmic Protein Extraction Kit (Beyotime Biotechnology, China) following the manufacturer's instructions.

### Statistical analysis

Statistical analysis was performed using SPSS version 22.0 (SPSS, USA). Student-t test or one-way ANOVA was used to compare the difference between groups. All the experiments were performed at least 3 times and each value was presented as mean±S.D. The relationship between NKILA expression and clinicopathological characteristics were analyzed by Chi-squared test, and survival analysis was performed using Kaplan-Meier curves and log-rank test. Cox proportional hazards model was used to analyze OS predictors. Difference was considered significant at a level of P < 0.05.

## Results

### NKILA is down-regulated in HCC and acts as an independent predictor of HCC patients' prognosis

**Figure 1:**
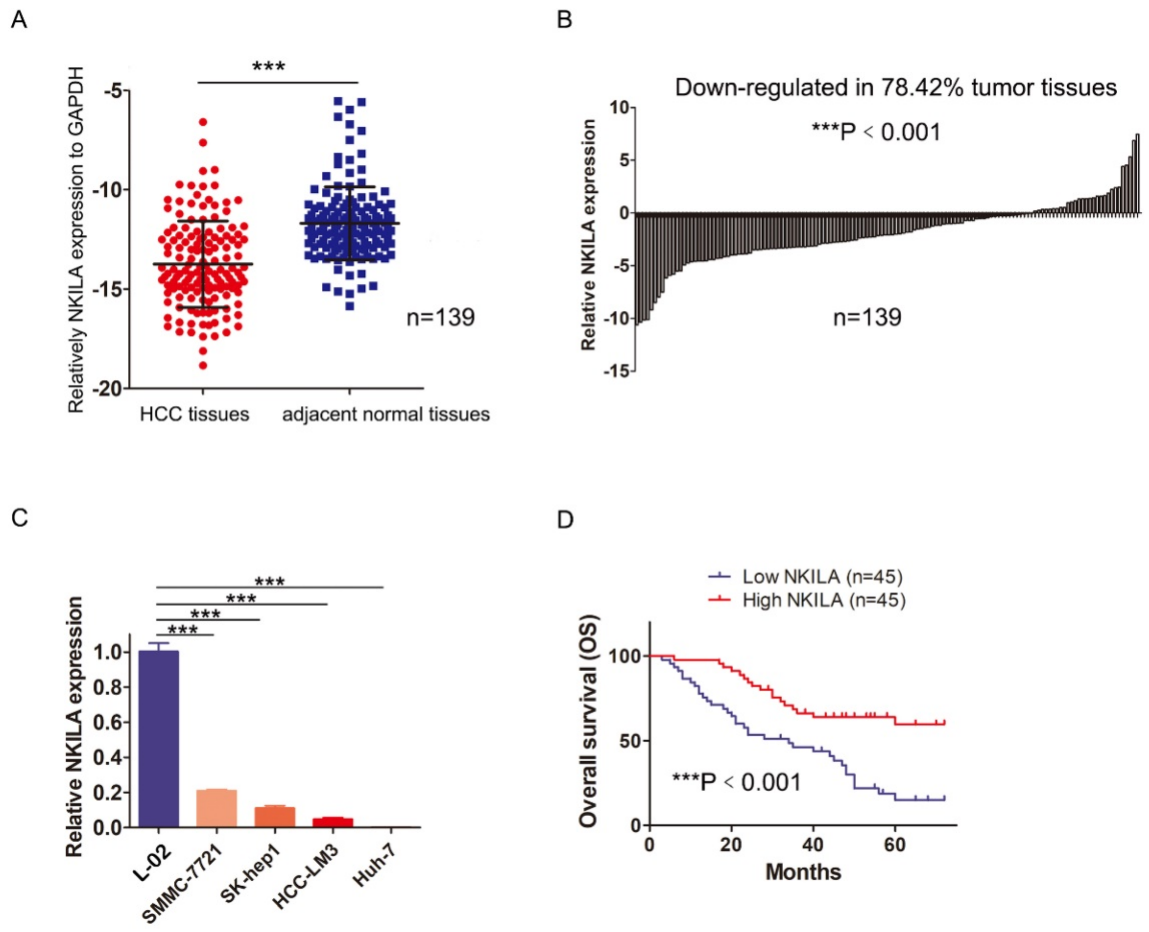
** NKILA is down-regulated in HCC and acts as an independent predictor of HCC patients' prognosis. (A)** The expression of NKILA in 139 pairs of HCC tissues and corresponding adjacent normal tissues was detected by qRT-PCR. **(B)** The expression of NKILA in HCC tissues was normalized to that of corresponding noncancerous tissues. The data was shown as log_2_(Fold change) = log_2_(T_NKILA_/N_NKILA_). **(C)** NKILA expression in human immortalized normal hepatocytes L-02 and four human HCC cell lines was detected by qRT-PCR. **(D)** Kaplan-Meier overall survival curves of 90 HCC patients with low and high NKILA levels. The data was presented as mean ± SD of three independent experiments. ***P < 0.001.

In order to assess the role of NKILA in HCC, we first measured the expression of NKILA in 139 pairs of HCC and corresponding adjacent normal tissues by qRT-PCR. As shown in Figure 1A, the expression level of NKILA significantly decreased in HCC tissues (P < 0.001). Compared with corresponding adjacent normal tissues, down-regulation of NKILA expression was observed in 78.42% (109/139) of HCC tissues (P < 0.001, Figure 1B). Moreover, the expression level of NKILA was remarkably lower in four human HCC cell lines than human immortalized normal hepatocytes L-02 (P < 0.001, Figure 1C).

To explore the clinicopathological significance of NKILA, 90 out of 139 patients were taken into analysis (49 patients with incomplete clinicopathological data or lost to follow-up within 2 years after surgery were excluded). As depicted in Table 1, chi-square analysis revealed that decreased NKILA expression in HCC was significantly associated with larger tumor size and positive vascular invasion. Kaplan-Meier curves and log-rank test showed that the overall survival (OS) of the patients with low NKILA expression was significantly shorter than those with high NKILA expression (P < 0.001, Figure 1D). The 5-year OS rates of patients with low and high NKILA expression were 15.6% and 60.0%, respectively. Moreover, as shown in Table 2, univariate and multivariate analysis indicated that NKILA expression (HR 0.325, 95% CI 0.181-0.582, P = 0.009) as well as AFP values and tumor differentiation grade, were independent predictors of OS in HCC patients.

In a word, NKILA is down-regulated in HCC tissues and cell lines, and serves as an independent predictor of HCC patients' overall survival.

### NKILA inhibits migration and invasion of HCC cells* in vitro*

**Figure 2:**
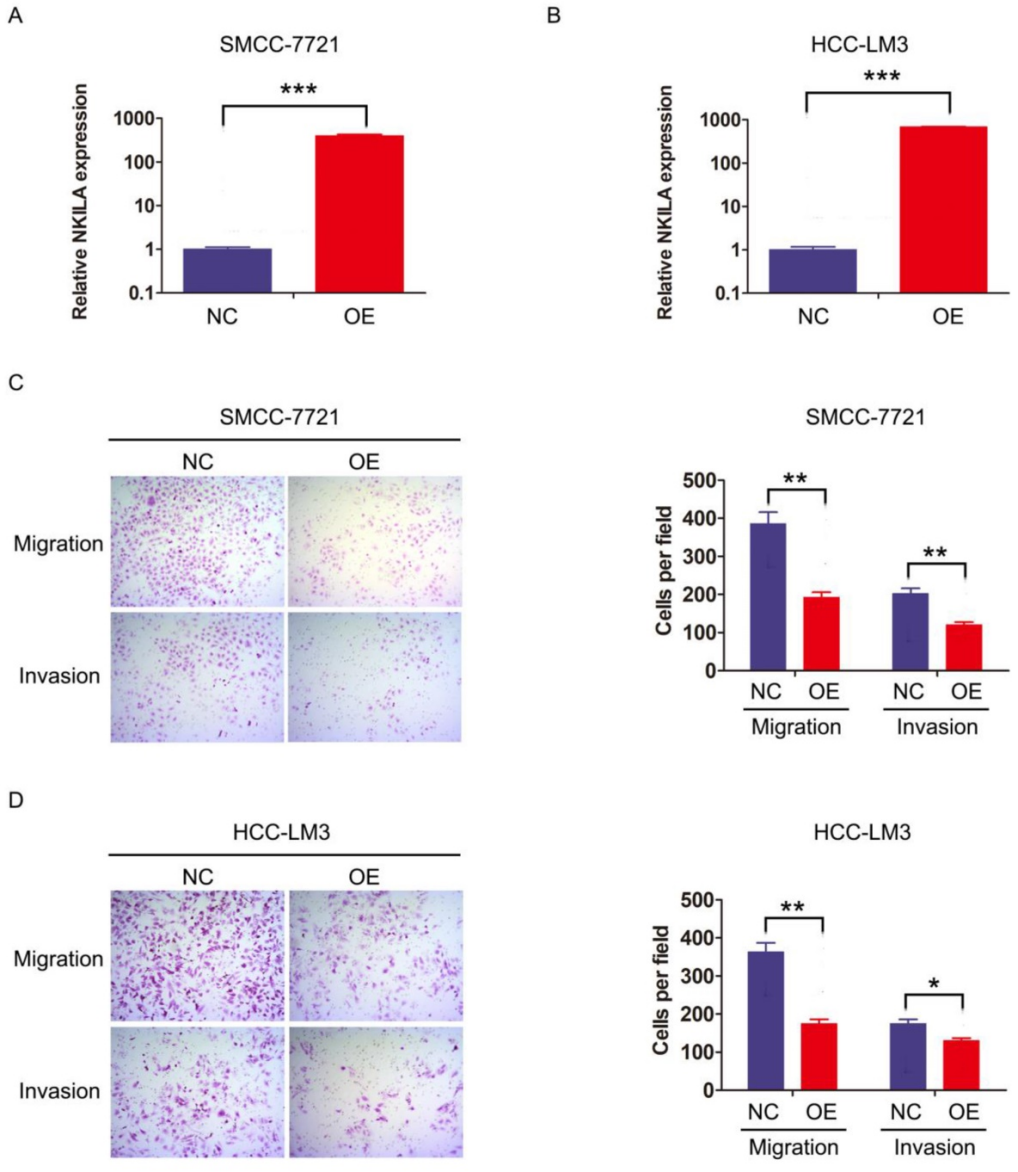
** NKILA inhibits migration and invasion of HCC cells* in vitro*. (A-B)** The expression of NKILA in SMMC-7721 and HCC-LM3 cells transfected with lentiviral vector expressing NKILA (overexpression group) or empty vector (negative control group) was detected by qRT-PCR. **(C-D)** The migration and invasion abilities of SMMC-7721 and HCC-LM3 cells in NKILA overexpression and negative control groups were detected by transwell assay, and images were obtained at 400× magnification. The data was presented as mean ± SD of three independent experiments. *P < 0.05, **P < 0.01, ***P < 0.001. OE, overexpression; NC, negative control.

We then investigated the function of NKILA on HCC cell biological behavior. SMMC-7721 and HCC-LM3 were transfected with lentiviral vector expressing NKILA or empty vector as negative control to establish stable expression cell lines (Figure 2A-2B). CCK-8 assay showed that overexpression of NKILA had little influence on the proliferation of these two HCC cell lines compared with negative control (Figure S1). Notably, as depicted in transwell assay, overexpression of NKILA significantly suppressed the migration and invasion abilities of SMMC-7721 and HCC-LM3* in vitro* (Figure 2C-2D).

### NKILA suppresses metastasis of HCC *in vivo*

**Figure 3:**
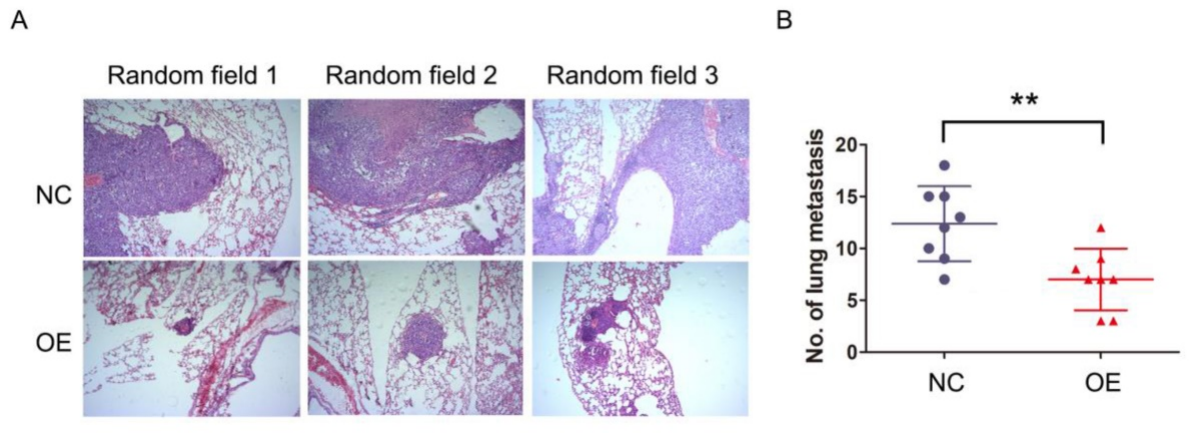
** NKILA suppresses metastasis of HCC *in vivo*. (A)** Representative images of lung sections stained by H&E in each group of SMMC-7721 cells. **(B)** The numbers of metastatic lesions in the lungs at 6 weeks after tail vein injection. **P < 0.01. OE, NKILA overexpression; NC, negative control.

The previous results demonstrated that NKILA inhibited migration and invasion of HCC cells *in vitro*, which raised a concern regarding whether this phenomenon would occur *in vivo* as well. SMMC-7721 cells stably expressing NKILA or empty vector as established before were injected into the tail vein of athymic nude mice to produce* in vivo* metastasis models. After 6 weeks, the lungs were examined by H&E staining. As shown in Figure 3A-3B, the number and size of metastatic nodules showed remarkable reduction in the overexpression group compared with negative control group. Taken together, overexpression of NKILA could also significantly suppress metastasis of HCC *in vivo*.

### NKILA prevents EMT via the inhibition of NF-κB/Slug pathway in HCC

**Figure 4:**
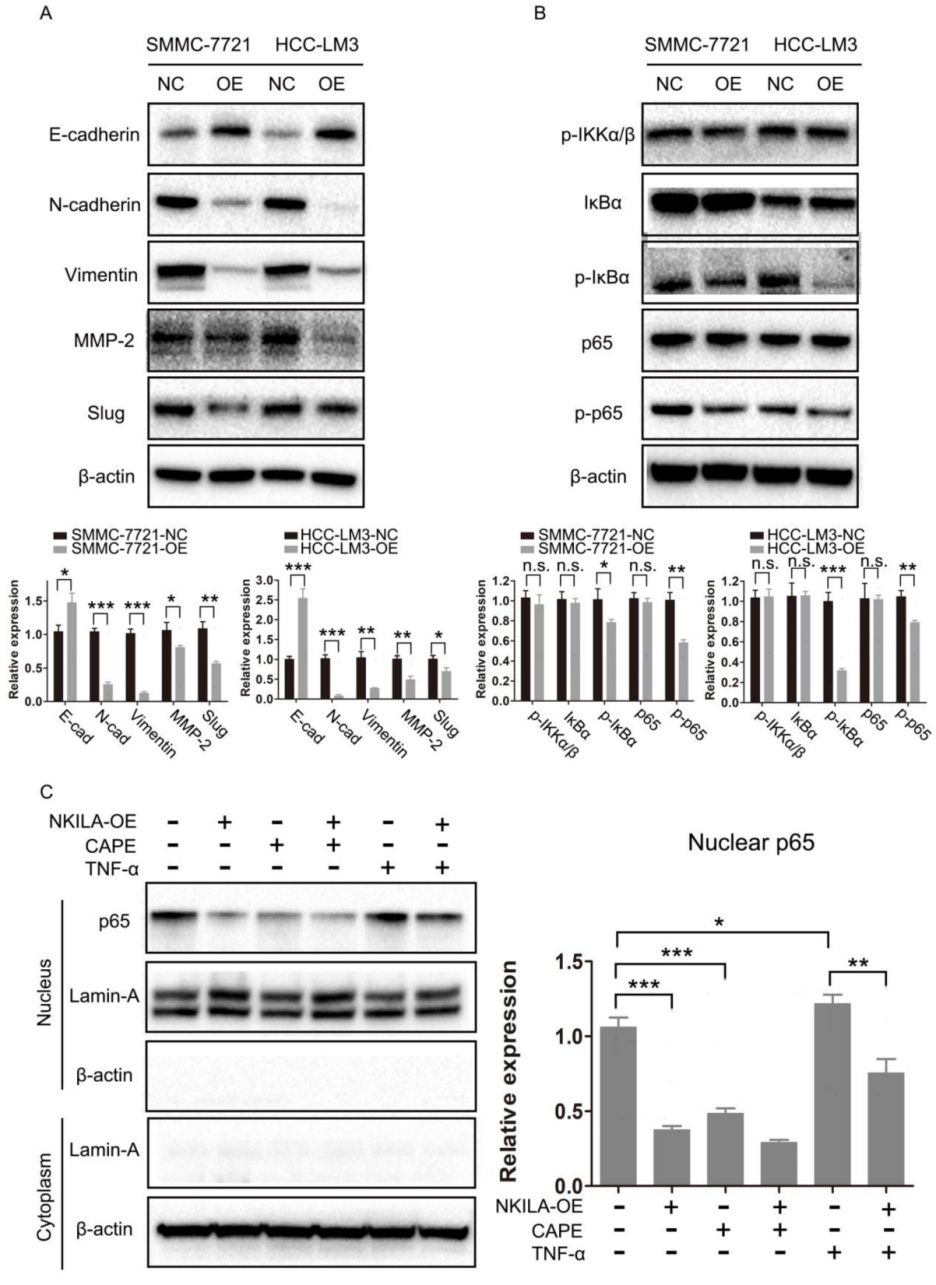
** NKILA prevents EMT via the inhibition of NF-κB/Slug pathway in HCC. (A-B)** EMT and NF-κB pathway related proteins of SMMC-7721 and HCC-LM3 cells in NKILA overexpression and negative control groups were detected by western blot. Relative semi‑quantitative analysis results were presented below. **(C)** Nuclear p65 of SMMC-7721 cells in NKILA overexpression and negative control groups treated with or without TNF-α or CAPE was detected by western blot. β-actin and Lamin-A were the loading control for cytoplasm and nuclear, respectively. Relative semi‑quantitative analysis results were presented (right panel). n.s., P > 0.05; *P < 0.05, **P < 0.01, ***P < 0.001. OE, overexpression; NC, negative control.

**Figure 5:**
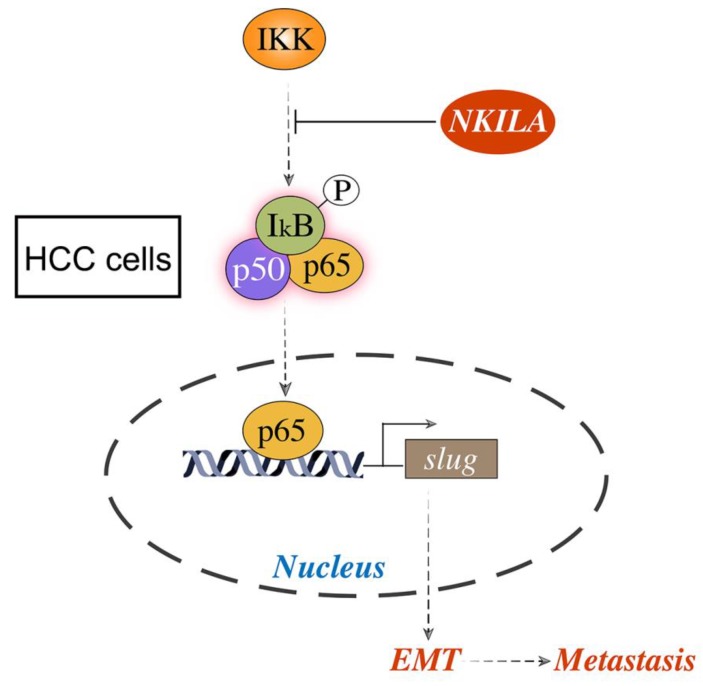
** Schematic model of the underlying molecular mechanism of NKILA on HCC metastasis.** NKILA inhibits HCC cells migration and invasion by hindering IKK induced IκB phosphorylation, p65 nuclear translocation and NF-κB activation, and subsequently suppressing Slug regulated EMT.

As EMT is closely related with metastasis of cancer cells 21, we further studied the connection of NKILA and EMT in HCC. As shown in Figure 4A, overexpression of NKILA led to a notable increase in the expression of E-Cadherin and a decrease in the expression of N-Cadherin, Vimentin, MMP-2 and Slug, indicating that NKILA could prevent EMT. NF-κB pathway was reported to play a pivotal part in regulating EMT of cancer cells 15, 16. Therefore, we next explored the role of NF-κB in NKILA induced EMT suppression. As depicted in Figure 4B, overexpression of NKILA had little effect on the expression of p-IKKα/β, but significantly decreased the expression of p-IκBα and p-p65. In addition, we tested p65 nuclear translocation to reflect the NF-κB activity. A classical NF-κB inducer TNF-α 22 and an inhibitor CAPE 23 were used to treat SMMC-7721 cells. As shown in Figure 4C, overexpression of NKILA or treated with CAPE remarkably inhibited p65 nuclear translocation compared with negative control, while treated with TNF-α promoted the translocation. Moreover, overexpression of NKILA could substantially withdraw the TNF-α induced nuclear translocation of p65. Collectively, NKILA prevents EMT via the inhibition of NF-κB/Slug pathway in HCC (Figure 5).

## Discussion

The activating invasion and metastasis is one of the six hallmarks of cancer 24. In HCC, intrahepatic and extrahepatic metastases are the major reasons leading to high recurrence rate and poor prognosis 25. This highlights the urgency of identifying high-risk patients in advance and establishing promising therapeutic targets for successful intervention. Nowadays, emerging studies have shown that lncRNAs are crucial for HCC metastasis and prognostic prediction. lncRNA GAS5 functions as a tumor suppressor in HCC metastasis through directly interacting with miR-182 and low level of GAS5 correlates with poor outcomes 26. lncRNA GALH promotes HCC metastasis via epigenetically regulating Gankyrin and acts as an independent unfavorable prognostic indicator 27. In this study, we focused on the newly identified lncRNA NKILA, evaluated the clinicopathological significance of NKILA in HCC patients and investigated the function and mechanism of NKILA on HCC cell biological behavior.

NKILA was first identified as an lncRNA upregulated by inflammatory cytokines via NF-κB Signaling in breast cancer. It interacts with NF-κB/IκB to form a stable complex, and directly masks phosphorylation motifs of IκB, thereby inhibiting IKK-induced IκB phosphorylation and NF-κB activation. Furthermore, NKILA suppresses breast cancer invasiveness, and NKILA expression was an independent predictor of breast cancer patients' prognosis 17. As NF-κB is a critical link between liver inflammation and cancer 28, 29, it is essential to figure out the precise role of NKILA and its interaction with NF-κB in HCC. To the best of our knowledge, this is the first study demonstrating the clinical significance of NKILA in HCC patients and investigating the function of NKILA on HCC cell biological behavior. Our results showed that the expression of NKILA was down-regulated in HCC tissues and decreased NKILA expression was significantly associated with larger tumor size and positive vascular invasion. NKILA reduction was an independent risk factor of HCC patients' poor prognosis. Moreover, overexpression of NKILA suppressed the migration and invasion of HCC cells both* in vitro* and *in vivo* by inhibiting the phosphorylation of IκB and NF-κB pathway. However, as the expression of NKILA was relatively low in HCC cell lines, we failed to do the knock-down experiments using siRNA and CRISPR/Cas9, therefore we only included the gain-of-function assays in this study.

Epithelial to mesenchymal transition (EMT) refers to a multistep, plastic and reversible morphologic transformation that enables epithelial cancer cells to acquire mesenchymal features, which has a significant role in tumor progression and metastasis 30. A set of transcriptional factors, including Snail, Slug, Twist, Zeb1 and Zeb2, orchestrate the EMT and subsequent migratory process 31. In this study, we demonstrated that the molecular mechanism underlying the effect of NKILA on HCC metastasis was through inhibiting Slug-EMT pathway. Overexpression of NKILA resulted in a notable increase in the expression of E-Cadherin and a decrease in the expression of N-Cadherin, Vimentin, MMP-2 and Slug. Our data contributed to a better understanding of the complex regulatory network of EMT.

In summary, NKILA is down-regulated in HCC tissues and cell lines, and positively correlates with HCC patients' overall survival. Moreover, NKILA inhibits HCC metastasis both *in vitro* and *in vivo* by hindering IκB phosphorylation and NF-κB activation, and subsequently suppressing Slug regulated EMT. These results indicate that NKILA might serve as an effective prognostic biomarker and a promising therapeutic target against HCC metastasis.

## Supplementary Material

Supplementary figure S1.

## Figures and Tables

**Table 1:** Correlation analysis between NKILA expression and clinicopathological parameters of HCC patients

Variables	NKILA expression		P value
	low	high	
**Age (years)**			0.829
≤55	17	18	
>55	28	27	
**Gender**			0.292
Female	6	3	
Male	39	42	
**HBV**			0.803
Absent	10	11	
Present	35	34	
**AFP (ng/ml)**			0.090
≤400	16	24	
>400	29	21	
**Cirrhosis**			0.455
Absent	12	9	
Present	33	36	
**Tumor Size (cm)**			0.006**
≤5	19	32	
>5	26	13	
**Tumor number**			0.822
=1	31	30	
>1	14	15	
**Vascular invasion**			0.003**
Negative	25	38	
Positive	20	7	
**Tumor differentiation**			0.393
Poor	28	24	
Moderate-Well	17	21	

HCC: hepatocellular carcinoma; HBV: hepatitis B virus; AFP: alpha fetoprotein. **P < 0.01.

**Table 2:** Univariate and multivariate analysis of clinicopathological parameters related to overall survival in HCC patients

Variable	Univariate		Multivariate	
	HR (95% CI)	P value	HR (95% CI)	P value
Age (≤55/ >55)	1.268 (0.721-2.231)	0.409	-	-
Gender (Female/Male)	0.858 (0.366-2.011)	0.725	-	-
HBV (Absent/Present)	0.901 (0.480-1.692)	0.746	-	-
AFP (>400/≤400)	3.949 (2.062-7.560)	0.000***	3.000 (1.549-5.810)	0.001**
Cirrhosis (Absent/Present)	0.918 (0.481-1.750)	0.795	-	-
Tumor Size (>5cm/≤5cm)	1.861 (1.077-3.215)	0.026*	-	-
Tumor number (=1/>1)	0.599 (0.344-1.043)	0.070	-	-
Vascular invasion (Positive/Negative)	2.779 (1.577-4.900)	**0.000**	-	-
Tumor differentiation (Moderate-Well/Poor)	0.375 (0.204-0.688)	0.002**	0.463 (0.252-0.852)	0.013*
NKILA expression (High/Low)	0.325 (0.181-0.582)	0.000***	0.454 (0.251-0.822)	0.009**

HCC: hepatocellular carcinoma; HBV: hepatitis B virus; AFP: alpha fetoprotein. *P < 0.05, **P < 0.01, ***P < 0.001.

## Abbreviations

AFP: alpha fetoprotein
CAPE: caffeic acid phenethyl ester
EMT: epithelial to mesenchymal transition
HBV: hepatitis B virus
HCC: hepatocellular carcinoma
H&E: hematoxylin and eosin
LncRNA: long noncoding RNA
NC: negative control
NF-κB: Nuclear factor-κB
NKILA: NF-κB interacting lncRNA
OE: overexpression
OS: overall survival
