# Supplementary material for: NKILA, a prognostic indicator, inhibits tumor metastasis by suppressing NF-κB/Slug mediated epithelial-mesenchymal transition in hepatocellular carcinoma
Source: Int J Biol Sci. 2020 Jan 1;16(3):495–503. doi: 10.7150/ijbs.39582 (PMC6990899; doi:10.7150/ijbs.39582)
Supplement: Supplementary file 1 — Supplementary figure S1. [file ijbsv16p0495s1.pdf]

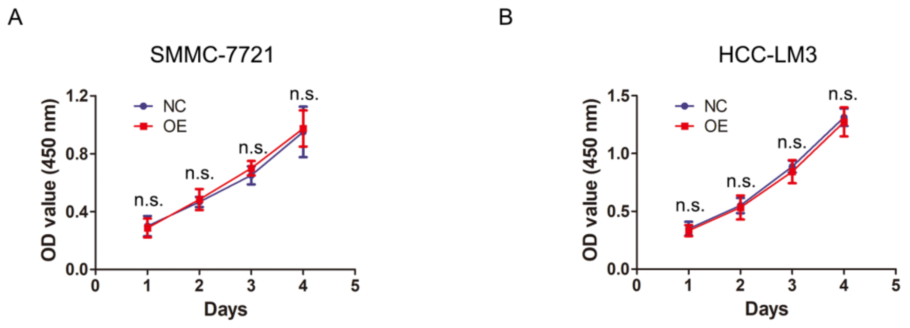

Figure S1. Overexpression of NKILA had little influence on the proliferation of HCC cells. (A-B) The proliferation abilities of SMMC-7721 and HCC-LM3 cells in NKILA overexpression and negative control groups were detected by CCK8 assays for four consecutive days. n.s., P＞0.05. OE, NKILA overexpression; NC, negative control.
